# Multi-Target Intense Human Motion Analysis and Detection Using Channel State Information†

**DOI:** 10.3390/s18103379

**Published:** 2018-10-10

**Authors:** Jialin Liu, Lei Wang, Jian Fang, Linlin Guo, Bingxian Lu, Lei Shu

**Affiliations:** 1 Key Laboratory for Ubiquitous Network and Service Software of Liaoning Province, School of Software, Dalian University of Technology, Dalian 116621, China; jialin.karen.liu@gmail.com (J.L.); fangjian@mail.dlut.edu.cn (J.F.); linlin.teresa.guo@gmail.com (L.G.); bingxian.lu@gmail.com (B.L.); 2 College of Engineering, Nanjing Agricultural University, Nanjing 210031, China; lei.shu@njau.edu.cn; 3 School of Engineering, University of Lincoln, Brayford Pool, Lincoln LN6 7TS, UK

**Keywords:** human motion detection, Channel State Information (CSI), multi-path effect, device-free, Support Vector Machine (SVM)

## Abstract

Intense human motion, such as hitting, kicking, and falling, in some particular scenes indicates the occurrence of abnormal events like violence and school bullying. Camera-based human motion detection is an effective way to analyze human behavior and detect intense human motion. However, even if the camera is properly deployed, it will still generate blind spots. Moreover, camera-based methods cannot be used in places such as restrooms and dressing rooms due to privacy issues. In this paper, we propose a multi-target intense human motion detection scheme using commercial Wi-Fi infrastructures. Compared with human daily activities, intense human motion usually has the characteristics of intensity, rapid change, irregularity, large amplitude, and continuity. We studied the changing pattern of Channel State Information (CSI) influenced by intense human motion, and extracted features in the pattern by conducting a large number of experiments. Considering occlusion exists in some complex scenarios, we distinguished the Line-of-Sight (LOS) and Non-Line-of-Sight (NLOS) conditions in the case of obstacles appearing between the transmitter and the receiver, which further improves the overall performance. We implemented the intense human motion detection system using single commercial Wi-Fi devices, and evaluated it in real indoor environments. The experimental results show that our system can achieve intense human motion detection rate of 90%.

## 1. Introduction

Device-free Passive (DfP) human detection has been a new technique to detect whether a human body appears in a certain area. Compared with active human detection, objects being detected do not need to carry any extra devices or wearable sensors which provides signals to be detected. Since in some cases, asking people to carry extra devices is inconvenient and unfriendly. Furthermore, it is difficult to ask people to carry devices under some intrusion detection scenarios, such as security monitoring in banks or enterprises. Therefore, there is an increasing demand on device-free passive human detection in some applications such as smart home, elderly care, assets protection and intrusion detection [1].

Nowadays, device-free passive human detection usually refers to techniques of two fields: camera-based and Wi-Fi-based. In the field of computer vision, researchers use image processing technology to analyze the video image collected by a surveillance camera. Camera-based methods can accurately extract human objects from images and spontaneously analyze their behaviors. This method has several shortcomings. First, the surveillance camera cannot work effectively in darkness or under weak light. Second, inappropriate deployment of surveillance cameras will cause many monitoring blind spots, which can be solved by increasing the number of cameras deployed. However, the hardware costs and the deployment complexity increase. Third, the biggest problem is that surveillance will cause privacy violation in places such as dressing rooms, restroom, bedrooms, as so on. These places are high-risk areas where incidents happen, including violence, illegal intrusion, and fall of elderly people. Due to various defects of camera-based technologies, Wi-Fi-based human motion detection techniques have emerged in recent years.

With the popularity of wireless networks, more and more wireless techniques have been applied. Wi-Fi has almost covered every corner of our life. It can be used not only for data transmission and wireless communication, but for sensing the environment as well. Using the already deployed wireless network topology for human motion-sensing and detection can save the cost of deploying specialized equipments and reuse the transmitted data at the same time, making fully use of the wireless resources. Therefore, wireless network provides us a useful means, which has great potential.

The CSI in wireless signal is physical layer information, which can depict the state of channel, reflecting the fine-grained characteristics in signal propagation process. The movement of object, especially the human motion, will have a great influence on the channel state in current environment. Leveraging this feature, we propose a method based on CSI for detecting intense human motion in complex scenarios. In this paper, we mainly focus on the indoor intense motion involving two targets whose motion range is relatively large, and the movement speed of the body and limb is high. The intense human motion has the following characteristics in general:

- Intense human motion with multi-targets usually involves multiple moving objects compared with human daily activities. There are interactions between one object and other objects. For example, simple human motions such as walking, squatting, bending over, and so on, involve a single object. Furthermore, more complex human motions such as hugging and shaking hands, involve objects no more than two. The intense human motions being detected in this paper such as fighting, involve the interactions between more than two objects.
- Compared with human daily activity, the movement pattern of intense human motion is more complex, irregular and difficult to predict. The actions are usually disorderly and intense, and the movement speed is much higher.

**Figure 1:**
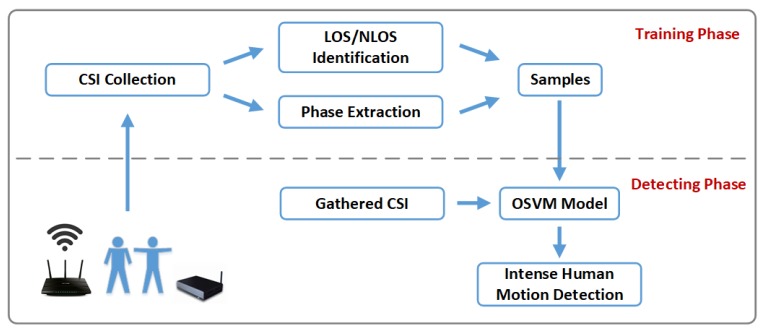
The framework of the intense human motion detection system.

Generally, if human motion with the characteristics above exists in the current indoor environment, we consider that intense human motion such as fighting happens. In real scenarios, there exists some furnitures and obstacles, which will cause influence to intense human motion detection. For these cases, we add LOS identification to our paper to improve the accuracy of human motion detection. We aim to realize human motion detection in complex scenarios using wireless physical layer information CSI. The framework of our system is shown in Figure 1. The definitions of the objective human motion and the complex scenario are as follows:

- The intense human motions being detected in this paper refers to violence and fighting behavior, involving the interactions between two objects with high speed limbs movement such as hitting, kicking and falling.
- The complex scenario refers to the case that obstacles exist between the transmitter and the receiver, and that is NLOS. These obstacles will have great influence on multi-path effect, and thus affect motion detection, and the influence to transmitted signals performs differently under LOS and NLOS conditions.

In summary, the contributions of our work are listed as follows:

- We conducted extensive experiments, finding out the pattern of the relationship between human motion and CSI variation. Then, we extract the feature from CSI to depict different human motion, and use machine learning methods to detect intense human motion from human activities.
- For complex scenarios, that is, there are obstacles between the transmitter and the receiver, we analyzed the signal variation difference under LOS and NLOS conditions, and then we identify whether the current wireless link status.
- We designed a intense human motion detection system which can be deployed on the Wi-Fi APs (Access Points). The system can monitor people’s behavior indoor in real time. It will alarm once it detects intense human motion.
- Our system does not require extra devices or equipments, and can achieve intense human motion detection in complex scenarios relying solely on existing Wi-Fi access point. Moreover, it can be used in private places where video cameras cannot be deployed, which makes up for the deficiencies that camera-based method has.

## 2. Related Work

In indoor environments, Wi-Fi signals experience reflection and scattering when transmitting from the transmitter to the receiver, which cause multi-path effect. The overlaid multi-path signals carry large amounts of information about the current features of the indoor environment. This provides the possibility of human motion detection using Wi-Fi signals. There are many existing work presenting different techniques of Wi-Fi-based device-free passive human detection and activity recognition, which is clearly different from our work.

### 2.1. Camera-Based Human Motion Detection

Camera-based human motion detection can automatically distinguish between normal human motion and intense human motion within the scope of video surveillance by detecting important characteristics such as the speed characteristics of the human body during movement, the dynamic characteristics of limb changes, and the motion trajectory. This technology has been widely used in intelligent video surveillance systems. Through the analysis of the image sequence captured by the video, a camera-based method can be applied to many applications including crowd counting [2], gesture recognition [3], target tracking [4], violence detection [5], and so on. Although camera-based methods have many advantages, they cannot be performed under weak light conditions or in places involving privacy issues.

### 2.2. Wi-Fi-Based Passive Human Detection

Wi-Fi-based passive human detection has attracted widespread concern in recent years. The conception of passive human detection in wireless environment was first proposed by Youssef, who implemented a Device-free Localization (DfL) system by analyzing signal variance to detect the change of the environment, and it can detect, tracking and identify targets without carrying any devices [6,7]. Besides, many research studies have realized passive human detection by leveraging the variance of Received Signal Strength Indicator (RSSI) on the receiver [8,9,10,11].

The fine-grained CSI has been used to realize passive human detection, achieving higher accuracy in recent years. Crowd counting has many applications in people’s daily lives such as crowd control, marketing research and so on. Wireless channel state can reflect the moving state of people and then researchers can count the number of people by analyzing the RSS and CSI [12,13,14,15,16,17]. In addition, passive human tracking using CSI can realize passive human detection and moving speed measurement, which can be further applied to security, elderly care and retail business [18,19,20,21,22,23]. For more subtle human signs, recent research works achieve fine-grained heartbeat and respiration detection using CSI or RF signals in a controlling setting [24,25,26,27,28,29]. Among them, the work [27,28] leverage the influence of human target’s chest displacement on the Fresnel zone and then complete the detection of respiration.

The goal of passive human detection is to detect whether someone is present in a certain region by various techniques. Compared with passive human detection, our work mainly focus on detecting the intense human motion with fast body movements, such as hitting, kicking and falling, involving multiple targets to further alarm and avoid the abnormal events like violence and school bullying.

### 2.3. Wi-Fi-Based Activity Recognition

Wi-Fi has been widely used in indoor localization in the past decade [30,31,32,33,34,35,36], and has been gradually used in activity and gesture recognition in recent years. Qifan et al. [37] and Kellogg et al. [38] first explore to use Wi-Fi RF signals to recognize different body or hand gestures. Adib et al. [39] implement tracking targets through wall using RF signals. More recently, Zhao et al. [40] implemented the first system that infers 3D human skeletons of multi-users from RF signals. but those signals are not accessible with commodity Wi-Fi devices and needs some extra devices such as USRP and FMCW, which are not universal. With the CSI tractable on commodity Wi-Fi devices [41], extensive applications of activity recognition appear based on CSI. Wang et al. [42] utilize the variance patterns of amplitude and phase of CSI to implement human fall detection. Wang et al. [43] build a model and use PCA method to extract the relationship between human activities and the variance of CSI to implement human activity recognition, and model-based activity recognition becomes a general trend nowadays [44,45]. Leveraging Wi-Fi to recognize slighter human gesture becomes possible. Ali et al. [46] and Chen et al. [47] implement keystroke recognition using CSI. Moreover, Wang et al. [48] and Melgarejo et al. [49] utilize Wi-Fi devices equipped with directional antennae to implement lip motion and sign language recognition. With the deepening of research, multi-person’s gesture recognition and tracking has made progress [50]. As the extension of activity recognition, behavior recognition now becomes an issue facing a number of challenges and attracts discussion about the state of the art and trends of CSI-based behavior recognition techniques [51,52].

All the work mentioned above can only handle the detection of human activities in predefined sets. However, the multi-target intense human motion detection in this paper is more complicated. Specifically, the intense human motion to be detected in this paper is disorderly. As an anomaly detection, it cannot be detected from various daily activities that have been previously defined or collected. In other words, a large number of complex human activities make the space for intense human motion detection problems larger.

## 3. LOS/NLOS Identification

Obstacles often exist between the sender and the receiver in the real indoor environment, making the direct transmitting path blocked in the current wireless link. We call this NLOS propagation. The variance of signals caused by human activities has different performance in the LOS and NLOS conditions. Thus, it is necessary to identify whether the current wireless link is under LOS or NLOS conditions.

Although LOS and NLOS conditions can be distinguished by measuring the signal transmission delay in theory, commodity wireless infrastructure often fails to do that due to limited bandwidth. Signals traveling along NLOS paths tend to behave more randomly compared with those along an LOS path [53]. Hence, from a statistical perspective, the distributions of received signal envelope differ under LOS and NLOS conditions due to varied extents of spacial randomness. We choose the skewness of CSI as the feature of LOS identification.

Skewness *s* is defined as:

$$s=\frac{E{\left\{x-\mu \right\}}^{3}}{\sigma^{3}},$$

where *x*, μ and σ denote the measurement, mean, and standard deviation, respectively. A positive (negative) skewness indicates that the measured data spread out more to the right (left) of the sample mean. We can transform Channel Frequency Response (CFR) in frequency domain collected from CSI samples into CIR in time domain using Inverse Fast Fourier Transform (IFFT). CIR can be denoted as:

$$h\left(t\right)=\sum_{n=1}^{N}\alpha_{n}\delta \left(t-\tau_{n}\right).$$

Therefore, the expression of skewness on CIR will be:

$$s=\frac{E[(|h\left(t\right)|-\mu_{\left|h\right|}{)}^{3}]}{\sigma_{\left|h\right|}^{3}}.$$

## 4. CSI-Based Human Motion Detection

**Figure 2:**
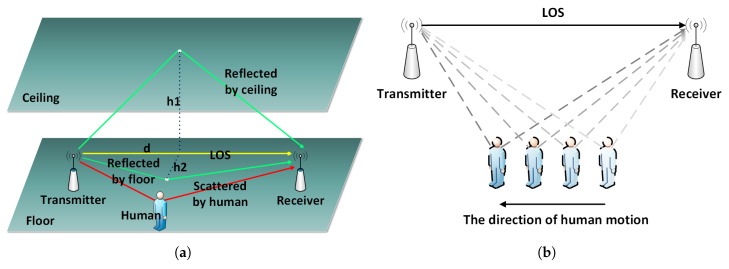
The indoor multi-path effects are affected by human body when people appear. (**a**) Shows the indoor signal propagation model. (**b**) Depicts the influence to wireless signal propagation paths caused by human motion.

In real transmissions, wireless signals are always affected by ceiling, floor and obstacles, which could lead reflection, scattering and diffraction before arriving at receivers. These effects attribute to multiple paths during the transmission, signals on different paths cause delay, fading and frequency diffusion, which give rise to the signal distortion. The distortion will overlay at the receivers and cause a total distortion which called multi-path effect. In a typical indoor environment, wireless signals always exist in the following types of paths: LOS, reflection, scattering, diffraction, and so on. The LOS is the main transmitting path of signals and have the strongest power. Due to the existence of walls, ceilings and floors, the signals would be reflected. Besides, the signals would also be affected when human beings appear in the room, which cause a diffraction path shown as Figure 2a.

The channel will keep relative stable when no human motion occurs in the current environment. However, as shown by the dashed lines in Figure 2b, as people move, the scattered signals generated by the human body change constantly, resulting in significant channel distortions of amplitude attenuation and phase shift. Therefore, we can recognize human motion by building a map between the pattern of signal variation and human motion.

Due to the time-varying channel, the multi-path effect also change with time. From the central limit theorem, it can be seen that the total signal distortion resulting from superposition of a large number of independent identically distributed signal distortions obeys a Gaussian distribution. Under the hypothesis of time-variant and multi-path propagation models, the channel can be described by the time-varying delay, intensity attenuation, and Doppler shift that occur over all signal propagation paths. The expression of the time-varying CSI is as follows:

$$h\left(\tau ,t\right)=\sum_{n}\alpha_{n}\left(\tau_{n}\left(t\right)\right)e^{-j2\pi f_{D_{n}}\tau_{n}\left(t\right)\delta \left(\tau -\tau_{n}\left(t\right)\right)},$$

where h(τ,t) represents the response of the channel at time *t* to the pulse sent at time t-τ. $\alpha_{n}\left(t\right)$, $\tau_{n}\left(t\right)$ and $f_{D_{n}}$ represent the time-varying signal attenuation factor, propagation delay, and Doppler shift for the *n*-th path.

In the field of indoor wireless communication, the model mentioned above can be simplified. Since the signal travels at a speed close to the speed of light, the transmission time of a signal packet is in the order of nanoseconds, so it can be assumed that the channel is time-invariant during transmission. In addition, the speed of motion of indoor objects is low, resulting in a Doppler shift of typically a few dozens of Hertz. Compared to the indoor delays of several tens of nanoseconds, the CSI can be simplified as follows:

$$h\left(t\right)=\sum_{n}\alpha_{n}\delta \left(t-\tau_{n}\right),$$

where h(t) represents the response of the channel at time *t* to the pulse sent at time 0. $\alpha_{n}$ and $\tau_{n}$ represent the signal attenuation factor and propagation delay for the *n*-th path.

The multi-path model provides a fine-grained description of the channel. When some paths obey certain regular changes, these changes are detected and the pattern of variation is extracted, which enables wireless sensing in specific areas. Next, we will discuss the CSI variation of amplitude and phase when human motion happens.

### 4.1. Human Motion and CSI Amplitude

The transmitter is equipped with one antenna and the receiver is equipped with three antennae. Therefore, the collected CSI can be further divided into three streams, each of which contains 30 subcarriers. So the CSI data can be depicted by the this form:

$$CSI^{1}=\left\{CSI^{1,1},CSI^{1,2},\cdots ,CSI^{1,30}\right\},$$

$$CSI^{2}=\left\{CSI^{2,1},CSI^{2,2},\cdots ,CSI^{2,30}\right\},$$

$$CSI^{3}=\left\{CSI^{3,1},CSI^{3,2},\cdots ,CSI^{3,30}\right\}.$$

**Figure 3:**
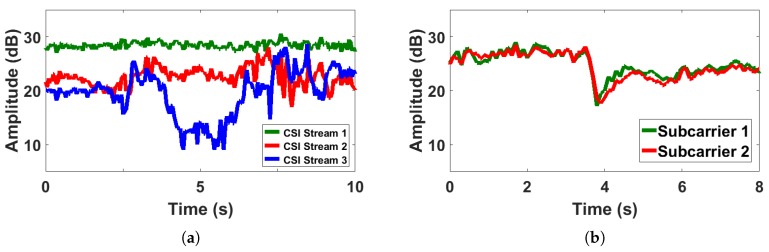
Human motion affects the CSI streams independently while affects different subcarriers in a similar way. (**a**) Shows the CSI variances of the first subcarrier in different streams when human motion occurs, while (**b**) shows the CSI variances of different subcarriers in one stream.

We conducted experiments to observe the CSI amplitude in different streams and subcarriers, respectively. As shown in Figure 3, it can be seen that the human motion during a period of time have different effects on different streams (Figure 3a), but have similar effects on different subcarriers (Figure 3b). Moreover, the effect of human motion on subcarriers with adjacent frequencies is more similar than that of subcarriers with farther frequencies. Thus, we use one subcarrier in one stream for analysis.

**Figure 4:**
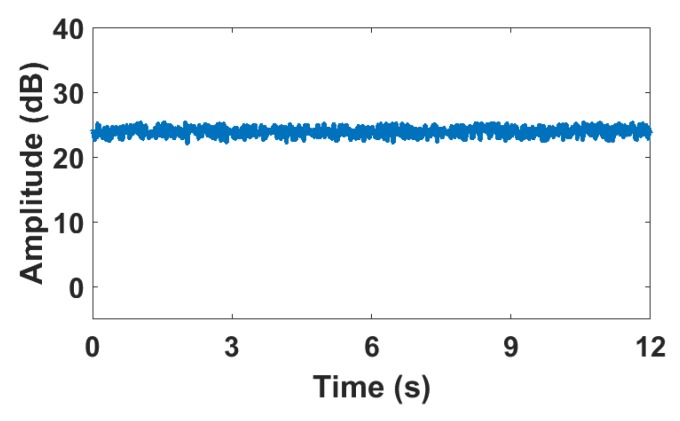
CSI amplitude variance under LOS condition when nobody appears.

**Figure 5:**
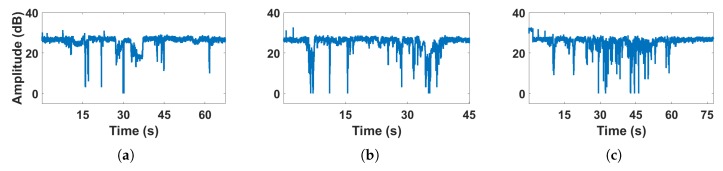
CSI amplitude of human motion activities (**a**) one person, daily activity; (**b**) two persons, daily activity; (**c**) two persons, intense motion.

Figure 4 shows the variation of CSI amplitude over time with no human motion under LOS conditions. It can be seen that the amplitude is stable near a certain value and there is a small range of fluctuations. Figure 5 shows the effect of different human motion on CSI amplitude under LOS conditions, respectively. Figure 5a shows a series of changes in CSI amplitudes caused by daily activities of a single person, including walking, sitting down, making phone calls, standing up, bending down, and squatting. Figure 5b shows that of two persons including handshaking, hugging, and chatting. Figure 5c shows that the two persons under the current environment have undergone a series of intense motion with higher speeds and larger body motion amplitudes, such as swinging arms, kicking, and wrestling.

The comparison of Figure 5a with Figure 5b shows that when the number of persons in the environment increases, the fluctuation of the CSI amplitude also increases to some extent. By comparing Figure 5a,b with Figure 5c, respectively, it can be found that when the two persons in the environment interact with each other producing a series of intense motion with high speed and large amplitude of limb movement, the CSI amplitude changes very dramatic. The most obvious observation is that continuous peaks occur. Even so, some of the multi-targeted intense human movements still cannot be effectively separated from other daily activities. We try to find some obvious signal features, but neither the variance nor the distribution of CSI amplitudes can find a obvious pattern that can be distinguished from other daily activities. Therefore, the CSI amplitude can only determine if there is a target moving in the current link. If human motion happens, the CSI amplitude fluctuates greatly; otherwise the CSI amplitude is relatively stable.

### 4.2. Human Motion and CSI Phase

As mentioned above, human motion can cause channel distortion and signal phase shift. Therefore, here we study the relationship between human motion and CSI phase information. Specifically, we use phase difference which carries the channel difference information of the two antennae at the receiver.

The measured phase $\hat{\phi_{i}}$ of CSI of *i*-th subcarrier can be computed as:

$$\hat{\phi_{i}}=\phi_{i}+2\pi f_{i}\Delta t+\beta +Z,$$

where $\phi_{i}$ is the true phase, Δt is the time lag at the antenna, β is an unknown constant phase offset, *Z* is some measurement noise, $f_{i}$ is the carrier frequency offset at the receiver.

**Figure 6:**
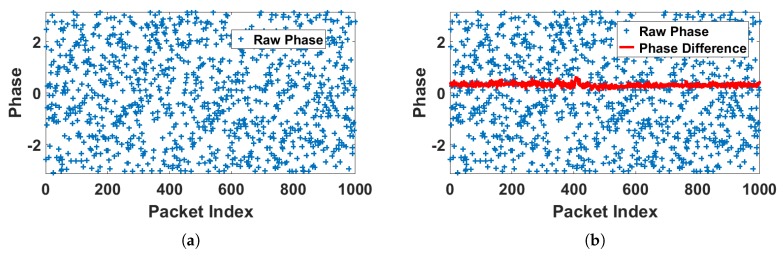
Raw phase and phase difference when no human appears in the current indoor environment. From (**a**) we can see the raw phases obtained by commodity wireless NIC distribute randomly which can not be used directly, while the raw phase can be calibrated phase difference as shown in (**b**).

Since it is difficult to measure and correct the synchronize error of the transmitter and the receiver, and the raw phases obtained by commodity wireless NIC distribute randomly as Figure 6a can not be used directly [42,54]. Thus, we use the phase difference which not only carries the phase information on two antennae, but also eliminate the error to some degree. Thus, the phase difference between two adjacent antennae can be calculated by:

$$\Delta \hat{\phi_{i}}=\Delta \phi_{i}+2\pi f_{i}\epsilon +\Delta \beta +\Delta Z,$$

where $\Delta \phi_{f}$ is the true phase difference, ϵ equals to the difference of time lag $\Delta t_{1}-\Delta t_{2}$ on antenna 1 and 2 respectively, Δβ is the unknown phase offset, and ΔZ is the noise. If the two receiving antennae are placed at a distance of half of a wavelength ($\frac{1}{2}\lambda$), then ϵ denotes the propagation time difference ($\Delta d\approx \frac{1}{2}\lambda sin\theta$) between the two antennae. The value of ϵ can be roughly estimated by the following formula:

$$\epsilon \approx \frac{1/2\lambda sin\theta }{cT}\leq \frac{1}{2Tf},$$

where λ is the wavelength, *f* is the center frequency, *c* is the speed of light, and *T* is the time interval between samples (approximately 50 nanoseconds in Wi-Fi), θ is the signal arrival direction. Since the frequency of Wi-Fi was chosen to be set at 5 GHz in the experiment, it can be approximately equal to zero. Therefore, the measured phase difference $\Delta \phi_{f}$ can be:

$$\Delta \hat{\phi_{i}}=\Delta \phi_{i}+\Delta \beta +\Delta Z.$$

From Figure 6b we can see that the randomly distributed raw phase can be calibrated by phase difference, which maintains on a stable level in a no human presence environment.

**Figure 7:**
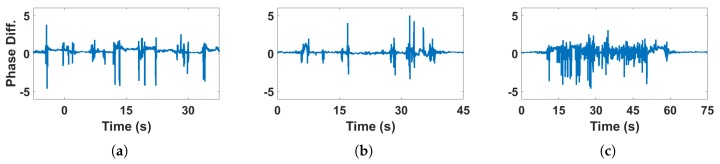
CSI phase difference of human motion activities under LOS condition (**a**) One person, daily activity; (**b**) Two persons, daily activity; (**c**) Two persons, intense motion.

Figure 7 shows the influence of human motion on CSI phase difference in LOS condition. Figure 7a shows the influence on CSI phase difference by the daily activities of one target such as walking, sitting, making phone call etc. Figure 7b shows influence of the daily activities of two targets, such as shaking hands, hugging, talking, and etc. Figure 7c shows influence of faster and intense motion like kicking and fighting by two targets in the same environment.

We can find that there is almost no difference on CSI phase when the number of targets is increasing, which approves the robustness of CSI phase difference to quantity of targets from comparison of Figure 7a and Figure 7b. However, continuous peaks appear on CSI phase difference when there are faster and more intense movements between two targets (by comparing Figure 7b and Figure 7c), which brings us the feasibility to detect intense human motion with this feature. From the view of CSI stream, since the phase difference is the sum of difference on each antenna [55], which means CSI phase difference is more representative than amplitude. We can see that intense motion leads to continuous peaks in the variance of phase difference. Thus, we use this characteristic as the feature to distinguish intense motion and daily activities.

### 4.3. Human Motion in LOS and NLOS Conditions

The observations mentioned above are the effects of human motion on CSI under LOS conditions. In real indoor scenarios, the wireless link between the transmitter and the receiver is often blocked by some displays, furnitures, and so on, which resulting in NLOS conditions. The emergence of NLOS will affect the quality of wireless link communication, resulting in the attenuation of wireless signals, which will affect the performance of various applications based on wireless technologies, such as the reduction of indoor positioning accuracy and the decrease in the accuracy of motion recognition. Therefore, it is necessary to identify whether the current environment is under LOS or NLOS conditions.

**Figure 8:**
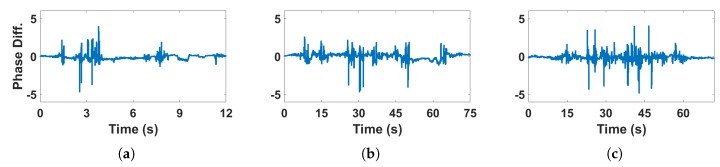
CSI phase difference of human motion activities under NLOS condition (**a**) One person, daily activity; (**b**) Two persons, daily activity; (**c**) Two persons, intense motion.

Figure 8 shows the influence of human motion on CSI phase difference in NLOS condition. The motion types are the same as in LOS condition described above. Compared with Figure 7, we can see that the influence caused by human motion is different in LOS and NLOS condition, the phase difference experience more fluctuate variance in NLOS condition than in LOS. Although we can detect intense motion by identifying continuous peaks in the variance of phase difference, we can not effectively detect the intense motion when both LOS and NLOS conditions exist at the same time. Therefore, it is necessary to identify LOS and NLOS condition at first.

### 4.4. Signal Preprocessing

Wireless signals, especially physical layer information CSI, are quite sensitive to environment variation. Therefore, preprocessing must be done. We could filter unrelated signal frequency component by setting threshold since signal variation caused by human movement is always in the range of 0–4 Hz. Otherwise, all the tiny moves are hidden in the normal human movements. Therefore, filtering signals under 4 Hz by band-pass filter is reasonable. We find that it is efficient to filter unrelated frequency and describe sharply human movement which provide a more advantageous signal for following detection.

### 4.5. Feature Extraction

We extract the following features from captured CSI to classify human motion: (1) Standard Deviation (STD), (2) Median Absolute Deviation (MAD), (3) Interquartile Range (IQR), (4) Signal Entropy. These features, which extracted from CSI amplitude and phase difference, are all set to be the input of OSVM.

### 4.6. OSVM Classifier

For the data in the training set that has only one type of positive sample (or negative sample) and no other types of samples, One-class Support Vector Machine (OSVM) is usually used for classification. At this point, the sorter needs to learn the boundary of the training set data. Since there is no two types of data, the max margin method in general SVM cannot be used. Using a nonlinear function, the SVM can project the sample points that cannot be linearly segmented in the original space into the high-dimensional feature space, and separate the two types of data with a “straight” hyperplane to obtain a decision boundary in the original space. OSVM have been widely used in anomaly detection and change detection.

In the practical scene, we usually only have good knowledge about the normal daily activity but do not know about the abnormal situation. Taking this into account, we use OSVM as our classifier. We divide the collected CSI into segments, marking the intense motion as the positive samples and the daily activities as the negative samples. By fully training the negative samples, we can get an OSVM model and feed the collected CSI to be detected into the model to implement intense human motion detection.

## 5. Evaluation

We conduct a series experiments to verify the proposed method. Based on a large amount of actual measurement data, we validate the CSI amplitude and CSI phase respectively, and use the experimental method to give suitable parameters for the model.

### 5.1. Experimental Setup

**Figure 9:**
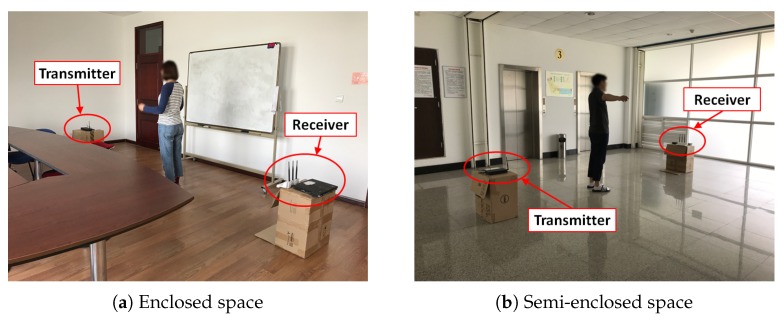
The scenario of human motion detection experiment.

**Figure 10:**
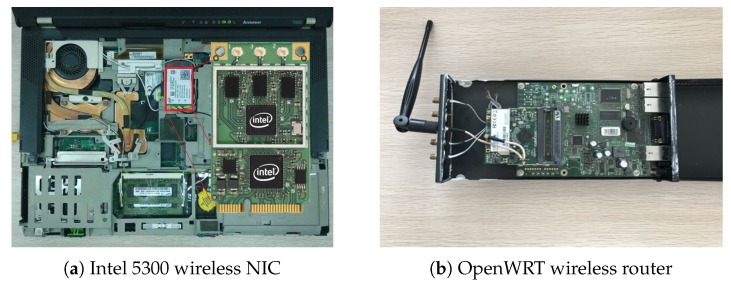
Experimental Hardware.

We test our system in an enclosed space and a semi-closed space, respectively. As shown in Figure 9, the enclosed space is a 7 m × 5 m meeting room and surrounded by concrete wall. There are wooden, plastic chairs and a 70 cm tall table in it. While the semi-closed space an 8 m × 8 m space with two sides of glass, one side of concrete wall and one side of stairs. The transmitter and the receiver are set in two ends of the space. The experimental hardware is shown in Figure 10. The sample rate is 30 packets per second.

### 5.2. Performance

#### 5.2.1. LOS/NLOS Identification

**Figure 11:**
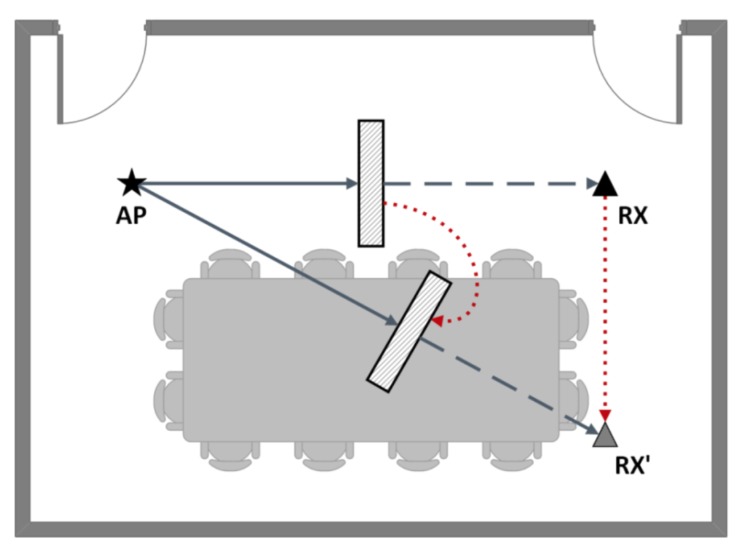
The scenario of LOS/NLOS identification.

To verify the validity of the LOS/NLOS identification based on the skewness of CIR distribution, we conducted the experiment in the above scenario. During the experiment, the AP at the transmitting end has a fixed position, and sends packets to the RX at the receiving end. The receiver gradually shifts from the initial RX position to the RX’ position, and we set 6 points of positions in total. We place a 1.5 m × 1 m × 3 cm metal board between the transmitter and the receiver to create NLOS condition as shown in Figure 11. As the receiver moves from RX to RX’, the position of the metal board is constantly adjusted while gathering data from NLOS path to ensure NLOS conditions. In this experiment, each measurement was conducted under stable state. We collect 2000 packets for each measurement, and in total we conduct 100 measurements. Each category of measurements include 50 LOS dominant conditions and 50 NLOS dominant conditions. We mainly focus on two metrics: (1) LOS Detection Rate $P_{D}$: The fraction of correctly identifying LOS conditions for all LOS cases. (2) False Alarm Rate $P_{FA}$: The fraction of wrongly identifying the LOS as NLOS condition for all NLOS cases.

**Figure 12:**
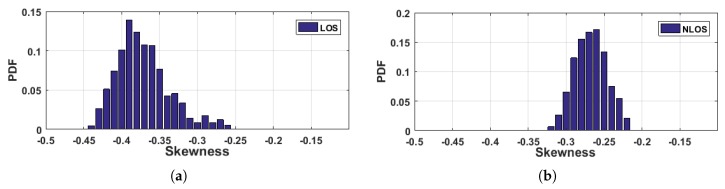
The distribution of CIR skewness under (**a**) LOS and (**b**) NLOS conditions.

**Figure 13:**
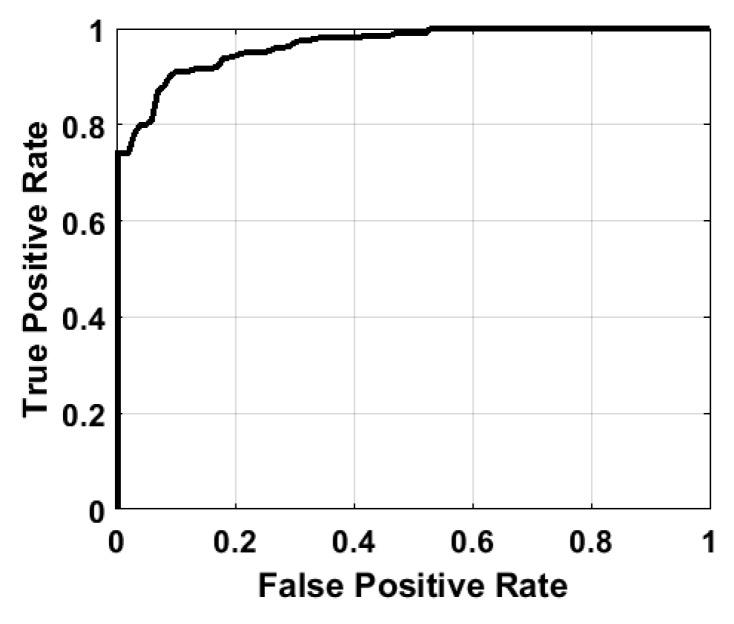
The indoor signal propagation model.

As illustrated in Figure 12, the skewness distribution of LOS condition is more negative than that of NLOS condition. To quantitatively evaluate the overall LOS identification performances of the two features, we plot the Receiver Operating Characteristic (ROC) curves of the two features in Figure 13. Given a constant false alarm rate of 10%, the LOS detection rates can achieve 91.56%

#### 5.2.2. Human Motion Detection

**Figure 14:**
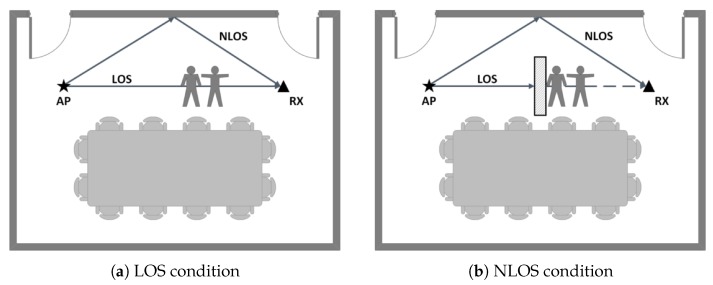
The scenario of human motion detection experiment.

We collected human motion data from four volunteers in the enclosed space and semi-enclosed space (Figure 14), recording the activities in LOS and NLOS conditions as follows:

(1) Walking, sitting, making phone call, standing up, squatting, bending over of one volunteer between the transmitter and the receiver.
(2) Walking, greeting, shaking hands, hugging of two volunteers between the transmitter and the receiver.
(3) Intense motion such as kicking, fighting of two volunteers between the transmitter and the receiver.

All the data generated from those motion above are continuous collected. We define motion in the first and the second data set as negative class, and motion in the third data set as positive class. We aim to detect all positive class from all motion. We assume that only motion in the first and the second data set happen in one day considering the real situation, and divide data into sample, each includes 150 packets. Samples of the first and the second data set are put into OSVM classifier for training, and samples from two classes are randomly picked to be tested into the model trained.

Two metrics are defined as follows: (1) Sensitivity (also defined as True Positive Rate, TPR) is defined as the percentage of correctly detected the intense human motion: TPR=TP/(TP+FN). (2) Specificity (also defined as True Negative Rate, TNR) is defined as the percentage of correctly detected the non-intense human motion: TNR=TN/(TN+FP).

Our evaluation are considered from four aspects:

- All the samples are not tagged with LOS or NLOS. Making all the negative samples as input of OSVM, and randomly selecting positive and negative samples as test set.
- LOS: Making all the negative samples as input and randomly selecting positive and negative samples as test set in LOS.
- NLOS: Making all the negative samples as input and randomly selecting positive and negative samples as test set in NLOS.
- All the samples are tagged with LOS or NLOS. Making all the negative samples as input of OSVM, making all the negative samples in LOS as input and randomly selecting positive and negative samples as test set.

**Figure 15:**
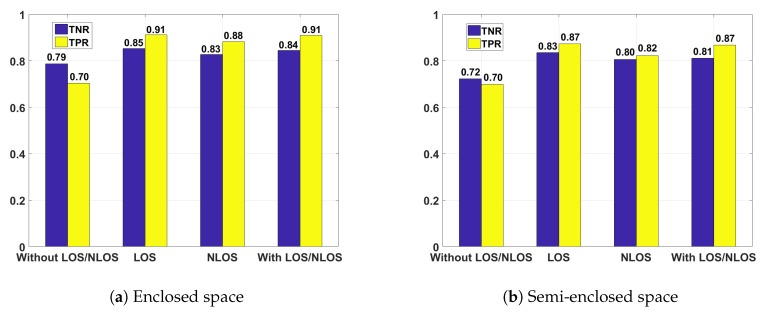
Performance results under different conditions.

The results in enclosed and semi-closed spaces are shown in Figure 15.

From Figure 15 we can see that the result is not good if samples are not tagged with LOS or NLOS. The main reason is that the positive samples under NLOS condition can not be well distinguished with the negative samples under LOS condition, resulting in the decrease in TPR and TNR. If we take LOS and NLOS condition into account respectively, it performs better in some extent. Through the comprehensive consideration and training of LOS and NLOS conditions, the detection rate of intense human motion can reach 90.89%, and the detection rate of normal human body movement can reach 84.43%.

Comparing the result of enclosed space and semi-closed space, we notice that both TPR and TNR in semi-closed space are lower than in enclosed space. The reason is that signals in semi-closed space are less affected by multi-path effect, therefore the influence on phase difference caused by intense motion is weaker.

#### 5.2.3. Performance Analysis

There are several factors that will affect the results when conducting the experiments. Here we analyze the influence of the height of the transmitter-receiver pair, the distance between the transmitter and the receiver, and the sample size on the experimental result.

**Figure 16:**
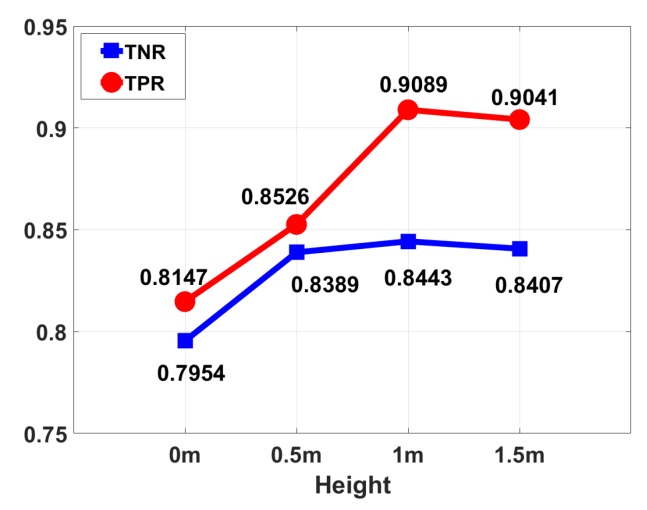
Performance results under different heights of TX and RX.

The height of the transmitter-receiver pair: In the experiment, the transmitter-receiver pair is placed at a height of 0 m, 0.5 m, 1 m and 1.5 m from the ground. Figure 16 shows the influence of different heights on the experimental result. From the histogram we can see that when the transmitter-receiver pair is placed at a low height (0 m and 0.5 m high from the ground), the experimental results are not satisfactory, whereas the results improved significantly when the transmitter-receiver pair is placed at a height of 1 m and 1.5 m. Considering the multi-path effect, the effect of human body on the reflection path caused by the floor is not significant when the transmitter-receiver pair is placed on the ground, resulting in poor results. Since the height of human body ranges from 1.5 m to 1.8 m, placing the transmitter-receiver pair at a height of 1–1.5 m is appropriate.

**Figure 17:**
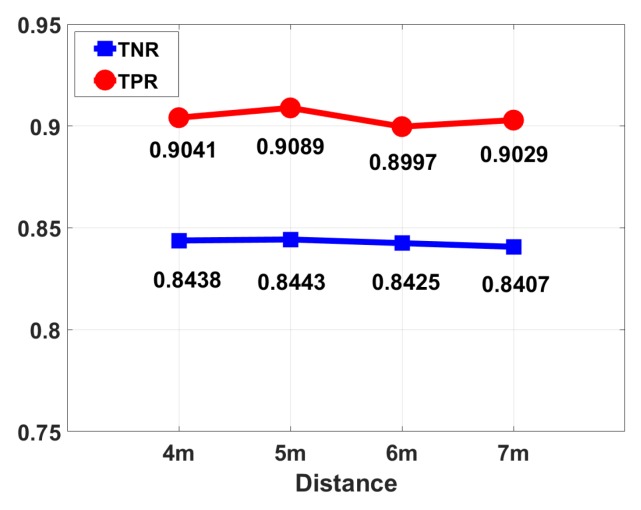
Performance results under different distances between TX and RX.

The distance between the transmitter and the receiver: Due to the limitation of the room space, the experiment measured the effect of the transmitter-receiver pair spacing 4 m, 5 m, 6 m, and 7 m on the experimental results. It can be seen from Figure 17 that the change in distance has little effect on the experimental results since the multi-path effect is not obviously affected.

**Figure 18:**
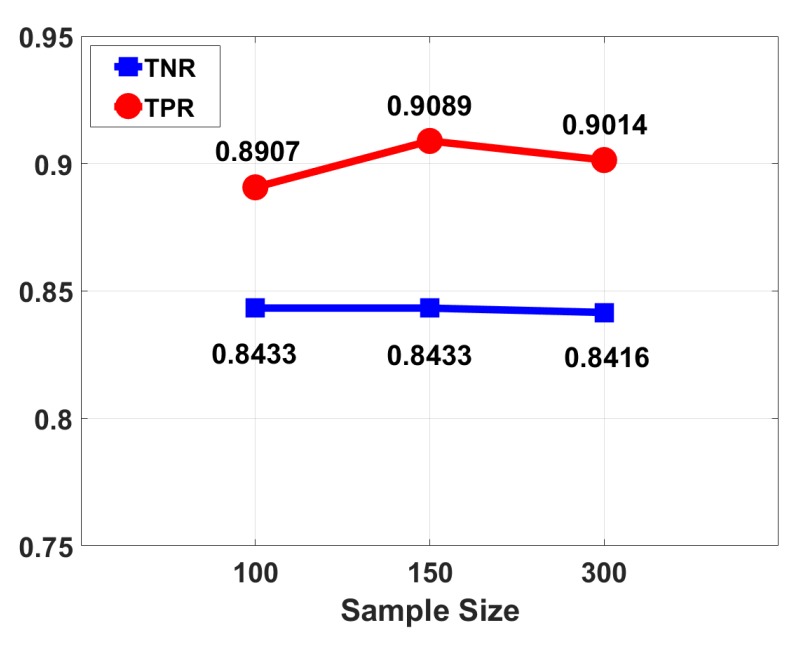
Performance results under different sample sizes.

The sample size: For the training sample size, we used 100, 150, 300 packets, i.e., 3 s, 5 s, 10 s in time, as training samples in the training phase. Through training and testing, the comparison results are shown in Figure 18. We can see that there is a slight decrease in detecting rate. The main reason is that the sample of 3 s is a bit short, and the actions that occur in a short time, such as squatting, will be confused with part of the intense human motion, causing wrong classification results.

## 6. Discussion

In this work, we realize multi-target intense human motion detection using CSI which is device-free and only uses commodity Wi-Fi devices. We discuss several interesting problems which are related to our work and worth further studying below.

Position-independent indicator: Our work achieves satisfactory accuracy when human motion happens on the direct path of the signal propagation. However, for the scenario that a large room equipped with one pair of transmitter and receiver (such as the distance between TX and RX is over 10 meters) and human motion occurs in the corner of the room, the detection rate will have an obvious decrease. Thus, we are looking for a position-independent indicator that measures the state of human motion no matter where human motion takes place in the indoor environment.

Multiple targets settings: Passive human motion detection for multiple targets faces big challenges. Our work can detect intense human motion between two targets from motion of no more than two persons. However, in real-life scenarios, human motion is diverse that is an intricate combination of many motion types. Therefore, how to detect the intense human motion involving more targets from the intricate human activities of groups of people requires further research.

People counting: For our further study, having a pre-estimation for the number of people in the indoor environment will be helpful for the intense human motion detection. Since there is a certain correlation between the probability of intense human motion and the number of people, we can further improve the performance of the system by counting the number of people in current indoor environment in advance.

Training-free human motion detection: Recent human motion detection using machine learning methods can achieve high performance, but at the cost of offline training in advance. Since offline training limits the application of the system in real life, we are looking for methods avoiding training from the perspective of signal propagation principles to implement real-time detection.

## 7. Conclusions

The work presented in this paper is a device-free passive multi-target intense human motion detection system which only uses the commodity Wi-Fi infrastructures. By analyzing the characteristics of the intense human motion distinguished with daily human activities, we find out the distinctive changing pattern of CSI phase difference between two different antennae influenced by intense human motion, and extracted features by conducting a large number of experiments. Moreover, we find the changing pattern performs differently in LOS and NLOS conditions. Thus, we address LOS and NLOS identification for further improvement. Experimental results demonstrate the performance of our system in different aspects. Our work can be further applied to security surveillance, property protection, and violence prevention. Although we implemented intense human motion detection, it still has some interesting problems worthy of further study, such as the location of motion happening relative to the transmitter-receiver pair, the motion of more than two persons, environment changes, and more effective machine learning algorithms. We are working on these problems and hope to obtain satisfactory results soon.

References
